# Effect of image registration on longitudinal analysis of retinal nerve fiber layer thickness of non-human primates using Optical Coherence Tomography (OCT)

**DOI:** 10.1186/s40662-015-0013-7

**Published:** 2015-02-12

**Authors:** Shuang Liu, Anjali Datta, Derek Ho, Jordan Dwelle, Daifeng Wang, Thomas E Milner, Henry Grady Rylander, Mia K Markey

**Affiliations:** Department of Biomedical Engineering, The University of Texas at Austin, Austin, TX 78712 USA; Department of Electrical and Computer Engineering, The University of Texas at Austin, Austin, TX 78712 USA; Department of Imaging Physics, The University of Texas MD Anderson Cancer Center, Houston, TX 77030 USA; Present address: Clinical Neuroscience Imaging Center (CNIC), Department of Neurology, Yale School of Medicine, New Haven, CT 06510 USA

**Keywords:** Retinal nerve fiber layer thickness, Glaucoma, Image registration

## Abstract

**Background:**

In this paper we determined the benefits of image registration on estimating longitudinal retinal nerve fiber layer thickness (RNFLT) changes.

**Methods:**

RNFLT maps around the optic nerve head (ONH) of healthy primate eyes were measured using Optical Coherence Tomography (OCT) weekly for 30 weeks. One automatic algorithm based on mutual information (MI) and the other semi-automatic algorithm based on log-polar transform cross-correlation using manually segmented blood vessels (LPCC_MSBV), were used to register retinal maps longitudinally. We compared the precision and recall between manually segmented image pairs for the two algorithms using a linear mixed effects model.

**Results:**

We found that the precision calculated between manually segmented image pairs following registration by LPCC_MSBV algorithm is significantly better than the one following registration by MI algorithm (p < <0.0001). Trend of the all-rings and temporal, superior, nasal and inferior (TSNI) quadrants average of RNFLT over time in healthy primate eyes are not affected by registration. RNFLT of clock hours 1, 2, and 10 showed significant change over 30 weeks (p = 0.0058, 0.0054, and 0.0298 for clock hours 1, 2 and 10 respectively) without registration, but stayed constant over time with registration.

**Conclusions:**

The LPCC_MSBV provides better registration of RNFLT maps recorded on different dates than the automatic MI algorithm. Registration of RNFLT maps can improve clinical analysis of glaucoma progression.

**Electronic supplementary material:**

The online version of this article (doi:10.1186/s40662-015-0013-7) contains supplementary material, which is available to authorized users.

## Background

Estimation of retinal nerve fiber layer thickness (RNFLT) is an important step in both glaucoma diagnosis and detection of glaucoma progression. RNFLT can be objectively and quantitatively measured by Optical Coherence Tomography (OCT). Because RNFLT maps measured by OCT are highly correlated with visual field loss [1-3], OCT can be used to assist in glaucoma diagnosis and longitudinal detection of glaucoma progression.

Studies suggest higher repeatability and reproducibility in measuring RNFLT of healthy and glaucomatous eyes with commercially available spectral-domain OCT compared to time-domain OCT instrumentation [4-6]. However, causes of measurement variability for example, manual placement of the scan circle by the instrument operator and patient eye rotation during successive measurements, remain problematic. Features of RNFLT such as temporal, superior, nasal and inferior (TSNI) quadrants averages, and 12 clock hour sector averages have been analyzed in clinical studies for glaucoma diagnosis [7-9]. In monitoring glaucoma progression, small changes of RNFLT features might be missed and false changes of RNFLT features might be detected because of misalignment of successive RNFLT maps. Therefore, accurate registration of maps recorded at different OCT imaging sessions is desired for assessment of glaucoma progression [10-12]. Recently, methods including tracking systems and scan alignments based on the optic nerve head (ONH) have been developed to improve image registration and RNFLT measurement reproducibility [12,13]. Some of the latest versions of commercially available spectral domain OCT software also incorporate methods to enable serial analysis of RNFLT changes. For example, the Spectralis OCT (Heidelberg Engineering, Heidelberg, Germany) uses a system to track eye movements and enable “real-time” registration. The OCT software package RTVue FD-OCT (Optovue, Inc., Fremont, CA) uses post-processing methods based on baseline images to enable registration [14]. Most of the recent registration methods for OCT scans are based on blood vessel structures or mutual information (MI) of fundus images [15-17]. Previous studies have shown that evaluation of RNFLT might be affected by variations in the position of the scan circle of measurements around the optic nerve that can compromise measurement reproducibility in eyes of healthy human subjects [18,19]. However, no study has been reported on whether image registration can improve longitudinal RNFLT evaluation in healthy eyes, and which RNFLT features may be more sensitive to misalignment of RNFLT maps recorded on different dates.

In this longitudinal study, we presented and compared an automatic algorithm based on MI and a semi-automatic algorithm based on log-polar transform cross-correlation using manually segmented blood vessels (LPCC_MSBV) for registration of RNFLT maps from a spectral domain OCT instrument of healthy non-human primates. We chose to investigate MI and LPCC_MSBV algorithms because they were demonstrated as two robust approaches for retinal image registration [20-23]. We evaluated changes in 17 different RNFLT features calculated from the RNFLT maps (all rings average, TSNI quadrants average and 12 clock hour sectors average) with and without registration over a 30-week time period.

## Methods

### Experimental design

Retinal nerve fiber layer (RNFL) imaging was performed on three macaque monkeys: two cynomolgus monkeys (macaca fascicularis), and one rhesus macaque monkey (macaca mulatta). One eye (OS) in each primate was followed over a period of 30 weeks during which weekly OCT imaging and measurement sessions were performed to assess the IOP and record RNFL thickness [24].

All studies performed in this work were done under the direction of The University of Texas Institutional Animal Care and Use Committee, which followed an approved protocol (#08013001), and adhered to the ARVO Statement for the Use of Animals in Ophthalmic and Vision Research. The OCT system utilized to image the primates is a custom-built tabletop research instrument Polarization Sensitive OCT (PS-OCT) with free-space optics constructed for the purpose of this study [24,25]. Comparison of this OCT system to RTVue and Cirrus OCT systems is shown in the Additional file 1: Table S1. The PS-OCT system uses a swept laser source (Santec, HSL 1000) with a 1 μm center wavelength and axial resolution of 12 μm. Lateral resolution is approximately 25 μm. Average incident power on the primate cornea was 1.13 mW. The head of the anesthetized primate was gently secured in a cradle with angular position controlled by two goniometers. Eye orientation was manipulated with sutures at the limbus to bring the ONH into the center of the field of view, and resulted in significant variation in the globe orientation between imaging sessions. Moreover, placement of the scan circle by the instrument operator was not repeatable between imaging sessions and introduced some translational misalignment in RNFLT maps.

**Figure 1:**
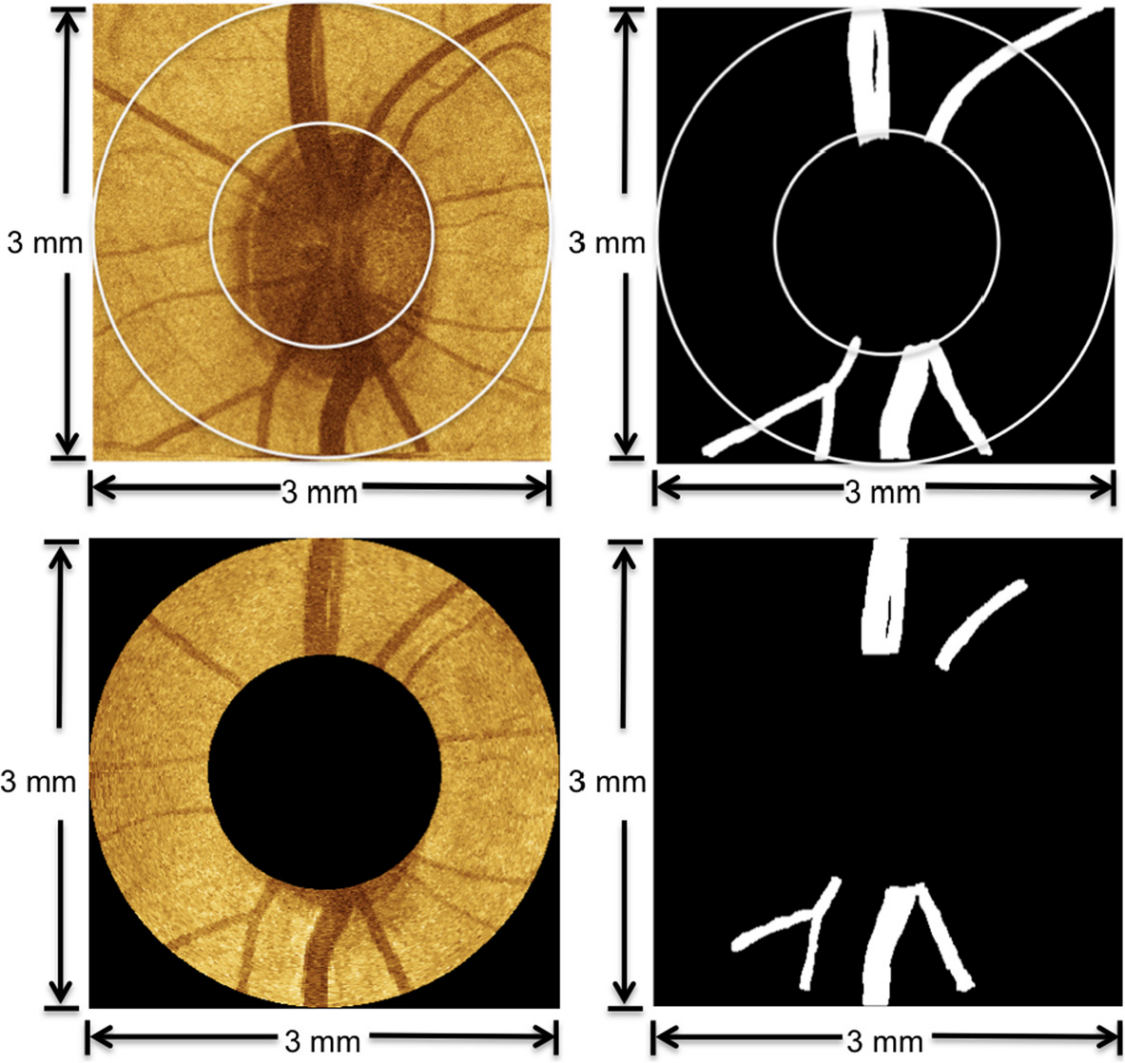
**Examples of reference image, target image, and blood vessel segmentation.** Images in the top row are derived from a raster scan fundus image (top, left) and manually segmented blood vessels (top, right). The raster scan fundus image is selected for each primate eye and the central area is used as the reference image for registration of RNFLT maps. Images in the bottom row are derived from a continuous ring scan fundus image (bottom, left) and manually segmented blood vessels (bottom, right). The continuous ring scan fundus image for each session is used as a target image to register RNFL thickness maps.

The left eye of each primate was imaged every week over a 30-week time period. Poor quality scans such as scans with A-scans affected by eye blinking or cases when the RNFL is out of the effective imaging depth were rejected by the instrument operator. There were 8 measurements for primate 1; 16 measurements for primate 2; 16 measurements for primate 3 over the 30 weeks selected for the analysis. Two scanning patterns were used to generate retinal maps. For each primate eye, one raster scan with best quality (minimum eye movement and best contrast) was performed on a 3 × 3 mm^2^ square area centered on the ONH. Each raster scan was comprised of 100 B-scans and each B-scan consisted of 256 A-scans. We created a raster scan fundus image by summing pixels of all the B-scan images and rescaled it to 256 × 256 pixels, and used it as the reference image for the respective primate eye. A second scanning pattern was a continuous ring scan pattern that contained 100 equally spaced ring B-scans centered on the ONH with ring diameters ranging from 1.5 mm to 3.0 mm. Each B-scan contained 100 A-scans. Data recorded from continuous ring scans were used to create an RNFL thickness map of each eye. Fundus images of continuous ring scans were created by summing pixels of all the B-scan images along the axial direction for registration purposes as target images (Figure 1).

### Retinal nerve fiber layer thickness map and feature calculation

**Figure 2:**
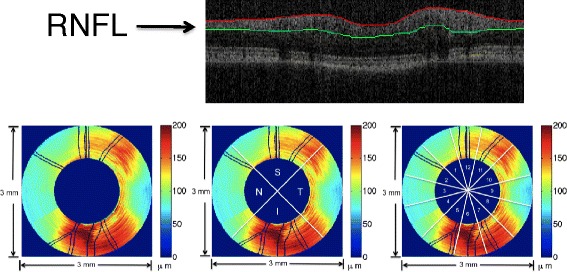
**Example of OCT B-scan image with segmented boundaries and feature parameter calculation of RNFLT map.** The upper panel is an example OCT B-scan image with segmented RNFL layer. The lower panel shows feature parameter calculation of RNFLT map of a primate left eye (OS). Lower Left is all-rings average of all 100 rings in the RNFLT map. Lower middle shows the temporal (T), superior (S), inferior (I) and nasal (N) quadrants in the RNFLT map. Lower right shows the 12 clock-hour sectors in the RNFLT map.

A LABVIEW software program (National Instruments, Austin, Texas) was implemented for the OCT system to automatically detect RNFL boundaries in each B-scan of continuous ring scans [25,26]. After RNFL boundary detection, an expert on OCT retinal image evaluation visually inspected the boundaries overlaid on each B-scan to correct any misidentified boundaries. RNFLT values were then imported into MATLAB (The Mathworks, Natick, MA) for RNFL feature calculation. The most widely used feature parameters were computed including the all-rings average thickness, TSNI quadrants average thicknesses, and each of the 12-clock hour RNFLT averages according to the OD clock-wise hours (Figure 2). Feature values were calculated on RNFLT maps before and after registration.

### Registration and evaluation method

**Figure 3:**
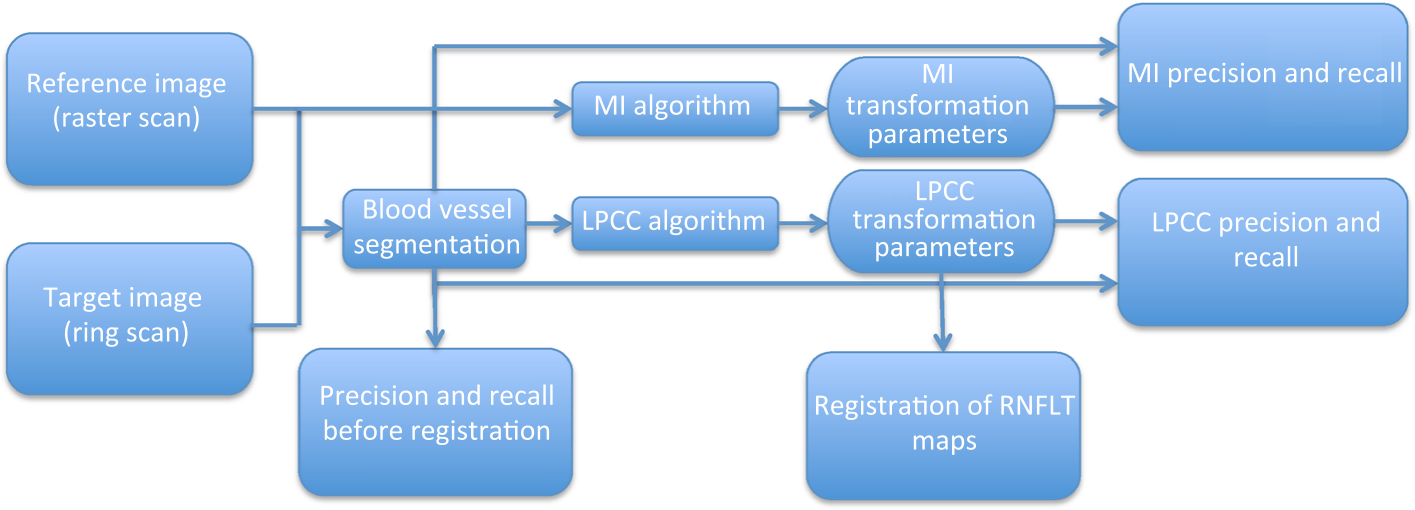
**Flowchart diagramming the application of MI and LPCC_MSBV algorithms.** Reference and target intensity images are used for the MI algorithm to find the transformation parameters (translation, rotation, scaling) to register the image pair. The manually segmented blood vessel images of the reference and target images are used for the LPCC_MSBV algorithm to find the transformation parameters (translation, rotation, scaling) to register the image pair. The precisions and recalls between manually segmented blood vessels in reference and target image pairs before registration (bottom left), after MI registration (upper right), and after LPCC_MSBV registration (lower right) are calculated. The LPCC_MSBV transformation parameters are used to register RNFLT maps.

One fundus image created from the raster scan was used as a reference image for each primate eye. All fundus images of continuous ring scans were target images and registered against this baseline image to ensure alignment of all RNFL thickness maps obtained with the continuous ring scan method. We applied two registration algorithms, MI and LPCC_MSBV) algorithms. The process of applying MI and LPCC_MSBV algorithms, and evaluation of precision and recall are shown in a flowchart (Figure 3). The original reference and target intensity images were used for the MI algorithm to determine the best transformation parameters to align the reference and target image pair. The manually segmented blood vessel images from reference and target images (e.g., Figure 1) were used for the LPCC_MSBV algorithm to find the best transformation factors to align the reference and target image pair. Precisions and recalls between manually segmented blood vessels of reference and target image pairs were used to evaluate the alignment between image pairs before and after registration. Details of the algorithms and evaluation process are described in the following sections.

#### Mutual information algorithm

We first used a MI algorithm to register RNFLT maps recorded on different days for the reference image [20-22]. The MI algorithm was performed on reference-target image pairs (Figure 3) and did not require segmentation of the blood vessels (Figure 1). The MI registration algorithm held the reference image fixed while the target image undergone transformations until images were registered. Linear transformation factors included x- and y-translation, rotation and scaling. The MI between a reference (A) target (B) image pair is defined as:

1 $$ \mathrm{M}\mathrm{I}\left(A,B\right)=H(A)+H(B)\hbox{--} H\left(A,B\right) $$

Where *H*(*A*) and *H*(*B*) are the Shannon entropies of the reference (A) and target (B) images, respectively, defined as

2 $$ \mathrm{H}(X)=-{\displaystyle \sum_{i=1}^Np\left({x}_i\right) \log p\left({x}_i\right)} $$

Where *p*(*x*_*i*_) is the probability of occurrence of the intensity value *x*_*i*_in the image. Similarly, *H*(*A*, *B*) is the joint Shannon entropy of images A and B, defined as

3 $$ \mathrm{H}\left(A,B\right)=-{\displaystyle \sum_{i,j}p\left(i,j\right) \log \left(i,j\right)} $$

Where *p*(*i*, *j*) is the joint probability of the image intensity pairs in the joint histogram of images A and B. Two images were considered registered when *MI* (*A*, *B*) had a maximum value with respect to the linear transformation parameters.

We performed the MI registration in two major steps: a coarse registration step followed by a fine registration step. For coarse registration, we translated the target image from −25 to 25 pixels (approximately 0.15 mm) in both x and y directions with an interval of 5 pixels (approximately 0.03 mm), and rotated the image −10 to 10 degrees in 2 degree intervals until maximum MI between the reference and target images was obtained. To reduce time for coarse registration, larger search intervals were used compared to those used subsequently in fine registration. Performing three transformations simultaneously helped to prevent the algorithm from stalling in a local maximum, which was more common if each type of transformation were to be performed separately. The coarse transformation parameters that provided the maximum MI were found and performed on the target image before fine registration.

In fine registration, all transformation parameters, translation, rotation and scaling were performed separately with smaller search intervals to maximize MI. We first did a scaling search for a scale factor between 0.85 to 1.15 with an interval of 0.01, then varied x-translation factor from −20 pixels to 20 pixels (approximately 0.12 mm) with an interval of 1 pixel (approximately 0.006 mm), then y-translation factor from −20 pixels to 20 pixels with an interval of 1 pixel, then rotation factor from −20 to 20 degrees with an interval of 0.1 degrees, and finally scaling again with search radius between 0.85 to 1.15 with an interval of 0.01.

#### Log-polar transform based cross-correlation algorithm

RNFLT maps recorded on different days were also registered to the reference image using LPCC_MSBV algorithm [23]. First, we segmented the blood vessels in the original intensity reference and target images manually. Blood vessel images were mapped into log-polar coordinates, so that rotation and scaling in the original image corresponded to translation in log-polar images. In polar space, the translational factor in the angle direction corresponded to a rotational factor. In addition, consider a scaling factor, a, between the images, such that (x, y) in one image maps to (ax, ay) in the other. In log space, (x,y) → (log *x*, log *y*)and (*ax,ay*) → (log *x* + log *a*, log *y* + log *a*), so translational shifts corresponded to scaling. Log-polar transformed images were then cross-correlated to determine the scaling and rotation factors. Because spatial-domain calculations, unlike frequency-domain computations, are not translation invariant, the log-polar transform and subsequent cross-correlation was completed for all possible choices of origin within a limited search area in the reference image. When the maximum cross-correlation was found, the choice of origin corresponded to translation and shifts in log-polar space corresponded to scaling and rotation. To speed-up the LPCC_MSBV algorithm, search for the maximum was completed at two resolution levels, using the parameters from the coarser level as an estimate of the parameters for the finer level.

Because the OCT instrument operator approximately centered the scan ring over the ONH before recording data, images are roughly aligned, and registration is achieved within a limited range of translation factors. Translation factors between image pairs were limited to 40 pixels (approximately 0.23 mm) to improve registration speed. For coarse registration, the images were subsampled to 1/4th the size, yielding 128 × 128 pixel images with 20 × 20 pixel (approximately 0.23 × 0.23 mm) search areas corresponding to x- and y-translation factors between −10 and 10 pixels (approximately 0.12 mm). Log-polar transforms of the target images were then cross-correlated with the log-polar transforms of the reference image.

To reduce computation time, all cross-correlations were calculated using the Fast Fourier Transform (FFT). The cross-correlation was linear in the scaling direction, but circular in the rotation direction. Therefore, the log-polar transforms of the images were zero-padded along the scaling axis, but not the rotation axis.

The optimal scaling, rotation, and translation parameters determined from these cross-correlations were then applied to the target image before fine registration. For fine registration, the 512 × 512 pixel blood vessel images were used, and the translation factors were limited to −4 and 4 pixels (approximately 0.02 mm). The linear transformation was computed as in coarse registration using log-polar transforms and cross-correlations.

#### Manual segmentation of blood vessels

Manual segmentation of blood vessel images was needed for two aspects of this study. First, blood vessel segmentation is a necessary pre-processing step for registration using the LPCC_MSBV algorithm. Second, we used the manual segmented blood vessels for calculation of precision, and recall of reference-target image pairs to evaluate performance of MI and LPCC_MSBV algorithms.

Segmentation of the blood vessels in both raster and continuous ring scan fundus images were completed manually. Using a tablet PC, the five widest blood vessels with branches in each fundus image were manually annotated.

#### Evaluation of the registration results

The MI and LPCC_MSBV algorithms were evaluated in terms of precision and recall between manually segmented reference, and target image pairs before and after registration. The overlapped scanning region of reference and target images was used for calculation of precision and recall. Precision and recall are defined as:

4 $$ precision=\frac{N_{TP}}{N_{TP}+{N}_{FP}} $$

5 $$ recall=\frac{N_{TP}}{N_{TP}+{N}_{FN}} $$

**Figure 4:**
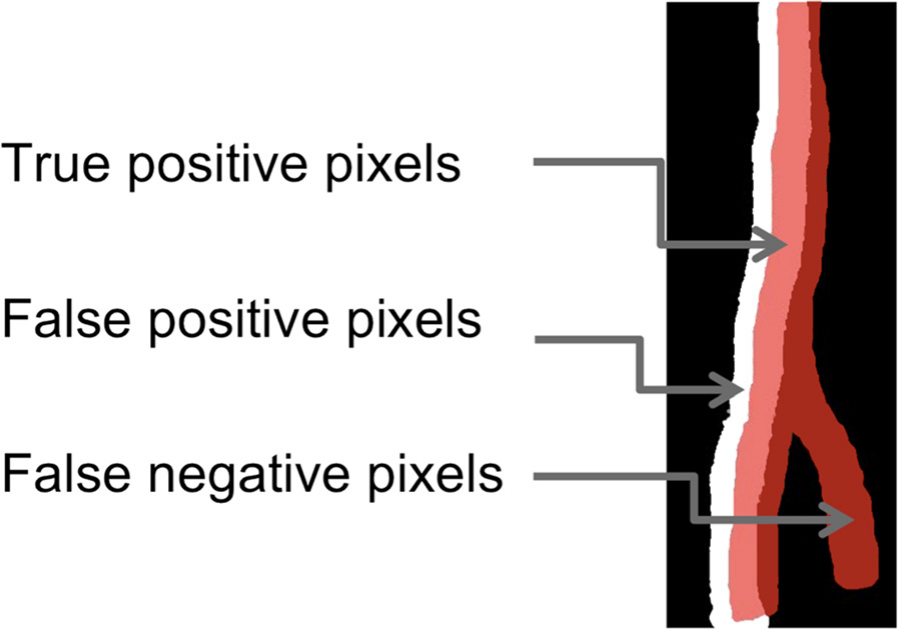
**True positive (TP); False positive (FP); False negative (FN) pixels for precision and recall calculation.** Red regions are the location of blood vessels in the reference image while white regions are the location of blood vessels in the target image. Blood vessel pixels that overlap in both reference and target images are marked as TP (light red). Blood vessel pixels in the target image, but not in the reference image are marked as FP (white). Blood vessel pixels in the reference image, but not the target image are marked as FN (dark red).

Where *N*_*TP*_ is the number of overlapping blood vessel pixels in reference and target images (true positives). *N*_*FP*_ is the number of blood vessel pixels in the target image, but not in the reference image (false positives). *N*_*FN*_ is the number of blood vessel pixels in the reference image, but not target image (false negatives) (Figure 4). The algorithm with better performance was used for registration of RNFLT maps.

### Statistical analysis

Linear mixed-effects models were used for longitudinal evaluation of estimated RNFL parameters to capture both the similarity (fixed effect) and variations (random effects) among the three primates. Linear mixed-effects models also provided unbiased analysis of balanced and unbalanced repeated-measurement data, which was consistent with our experiment design. We used the nlme package (R package version 3.1-104) [27] of R statistical programming language (v2.13.10 07/08/2011; http://www.R-project.org/, R Development Core Team, 2011, R Foundation for Statistical Computing, Vienna, Austria) and R studio (v0.94, 06/15/2011, RStudio, Inc.) for implementing the linear mixed-effects models.

We first evaluated whether the precision and recall calculated for registered image pairs by MI and LPCC_MSBV algorithms were significantly improved compared with no registration. We also used the precision and recall of image pairs registered by MI and LPCC_MSBV algorithms to compare the performance of these two algorithms. We used the following linear mixed effects model to evaluate the significance for the pairwise comparisons:

6 $$ {\mathrm{T}}_{\mathrm{i},\mathrm{t}}={\mathrm{T}}_{\mathrm{avg}}+{\mathrm{b}}_{\mathrm{i}}+\gamma \times re{g}_{\mathrm{i},\mathrm{t}}+{\varepsilon}_{\mathrm{i},\mathrm{t}} $$

Where T_i,t_ is precision or recall of the i^th^ primate control eye on day *t* since the beginning of the study, T_avg_ is the mean precision or recall across all the eyes. *b*_*i*_ is a random effect representing the deviation from T_avg_ for the i^th^ primate eye, normally distributed with zero-mean and standard deviation *δ*_*b*_; *reg*_*i,t*_ is a binary variable representing with (*reg*_*i,t*_ = 1) or without registration (*reg*_*i,t*_ = 0) for the i^th^ primate control eye on day t when the model was used for comparison of precision and recall, with and without registration. When the model was used for comparison of precision and recall of image pairs registered by MI and LPCC_MSBV algorithms, *reg*_*i,t*_ is a binary variable representing the algorithm used for the i^th^ primate control eye on day t (*reg*_*i,t*_ = 0 for MI algorithm; *reg*_*i,t*_ = 1 for LPCC_MSBV algorithm). γ is the slope for *reg*_*i,t*_; *ε*_*it*_ is a random effect representing the deviations in precision or recall on day *t* of the i^th^ primate eye from the mean precision or recall of the i^th^ primate eye, and normally distributed with zero-mean and standard deviation *δε*.

We investigated whether registration will affect the evaluation of RNFL thickness over time in this longitudinal study for healthy eyes. The following linear mixed effects model was applied,

7 $$ {\mathrm{RNFLT}}_{\mathrm{i},\mathrm{t}}=\left({a}_1+{\beta}_i\right)+{a}_2\times \mathrm{t}+{\xi}_{\mathrm{i},\mathrm{t}} $$

In the mixed effects model, RNFLT_i,t_ is a feature value in RNFLT maps of the eye of the i^th^ primate on day t since the beginning of the study. The intercept α_1_ and the mean slope α_2_ for number of days *t* are fixed effects. The random effect is the intercept β_i_ for i^th^ primate, which is normally distributed with zero-mean and standard deviation δ. ξ_i,t_ is the random error component for the i^th^ eye on day t, and assumed to be normally distributed with a mean of zero and standard deviation δ_e_.

## Results and discussion

### Comparison of MI and LPCC_MSBV algorithms

**Figure 5:**
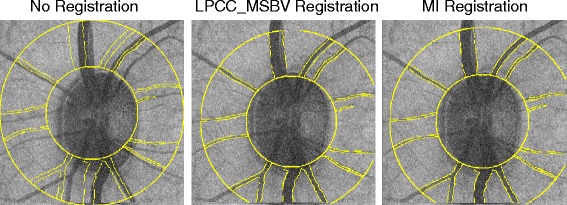
**Overlap of the reference and target images.** The reference image is shown as the gray scale intensity image. The target image is shown as transparent yellow lines in order to clearly demonstrate the overlap area. The overlap of the two images is shown before registration (left), after LPCC_MSBV registration (middle) and after MI registration (right).

**Figure 6:**
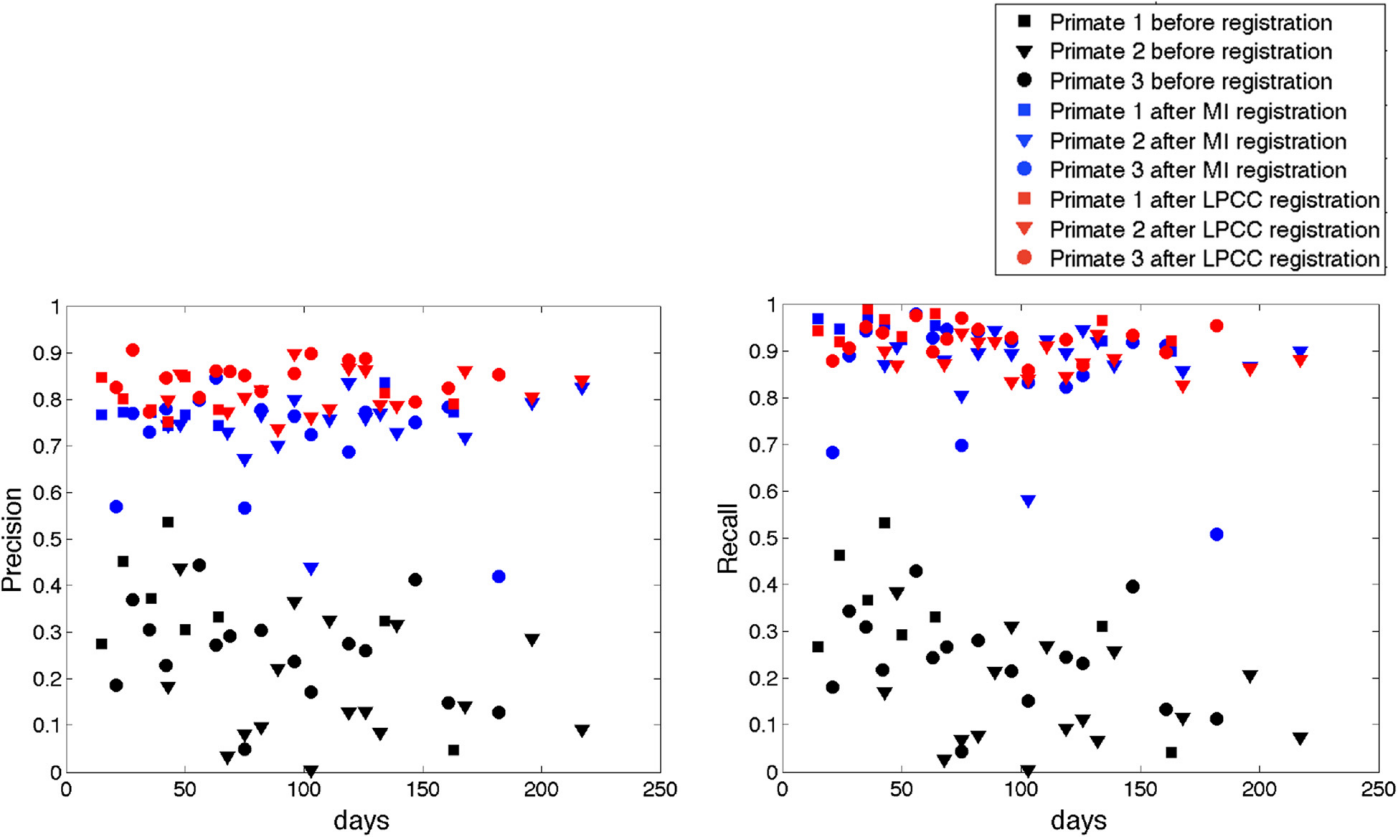
**Precision (left) and recall (right) before (black) and after registration by MI (blue) and LPCC_MSBV (red) algorithms.** Precision and recall following registration by both MI (blue) and LPCC_MSBV (red) algorithms are significantly better than values before (black) registration (p < <0.0001). Precision of the LPCC_MSBV (red) algorithm is significantly higher than that of the MI (blue) algorithm (p < <0.0001). Recalls of LPCC_MSBV and MI algorithms are not significantly different (p = 0.0571).

One example of overlap of the reference image and target image before and after MI and LPCC_MSBV registration is shown in Figure 5. By visual inspection, we can see that the overlap of the reference and target images is improved after both MI and LPCC_MSBV registration. Precision and recall were used to evaluate quality of registration results before and after application of MI and LPCC_MSBV algorithms (Figure 6). We used the linear mixed effects model described in Equation 6 to compare precision and recall values before vs. after registration and precision and recall values after registration by MI algorithm vs. LPCC_MSBV algorithm. Precision and recall following registration by either the MI or LPCC_MSBV algorithms were significantly better than that before registration (p < <0.0001, Tables 1 and 2). Thus, either the MI or LPCC_MSBV registration algorithm could significantly improve alignment of reference and target images. Precision of the LPCC_MSBV algorithm was significantly higher than that of the MI algorithm (p < <0.0001, Table 3). Recalls of the MI and LPCC_MSBV algorithms were not significantly different (p = 0.0571, Table 3). Inasmuch as the results suggest that the LPCC_MSBV algorithm performs slightly better than the MI algorithm on recorded primate images, we used the LPCC_MSBV algorithm to register maps for analysis of RNFLT versus time.

**Table 1:** **Results of comparing precision and recall values of before registration (** ***reg***_***i,t***_ **= 0) vs. after MI registration (** ***reg***_***i,t***_ **= 1)**

**Evaluation**	**Linear mixed effects model coefficients and p values**			
	**Intercept**	**p value for intercept**	**Slope of** ***reg***_***i,t***_	**p value for slope**
**Precision**	0.2488	<<0.0001	0.4972	<<0.0001
**Recall**	0.2332	<<0.0001	0.6588	<<0.0001

**Table 2:** **Results of comparing precision and recall values of before registration (** ***reg***_***i,t***_ **= 0) vs. after registration by LPCC_MSBV algorithm (** ***reg***_***i,t***_ **= 1)**

**Evaluation**	**Linear mixed effects model coefficients and p values**			
	**Intercept**	**p value for intercept**	**Slope of** ***reg***_***i,t***_	**p value for slope**
**Precision**	0.2453	<<0.0001	0.5829	<<0.0001
**Recall**	0.2323	<<0.0001	0.6905	<<0.0001

**Table 3:** **Results of comparing precision and recall values of registration by MI algorithm (** ***reg***_***i,t***_ **= 0) vs. LPCC_MSBV algorithm (** *reg*_*i,t*_ **= 1)**

**Evaluation**	**Linear mixed effects model coefficients and p values**			
	**Intercept**	**p value for intercept**	**Slope of** ***reg***_***i,t***_	**p value for slope**
**Precision**	0.7388	<<0.0001	0.0857	<<0.0001
**Recall**	0.8871	<<0.0001	0.0318	0.0571

### Analysis of RNFL thickness over time with and without registration

**Figure 7:**
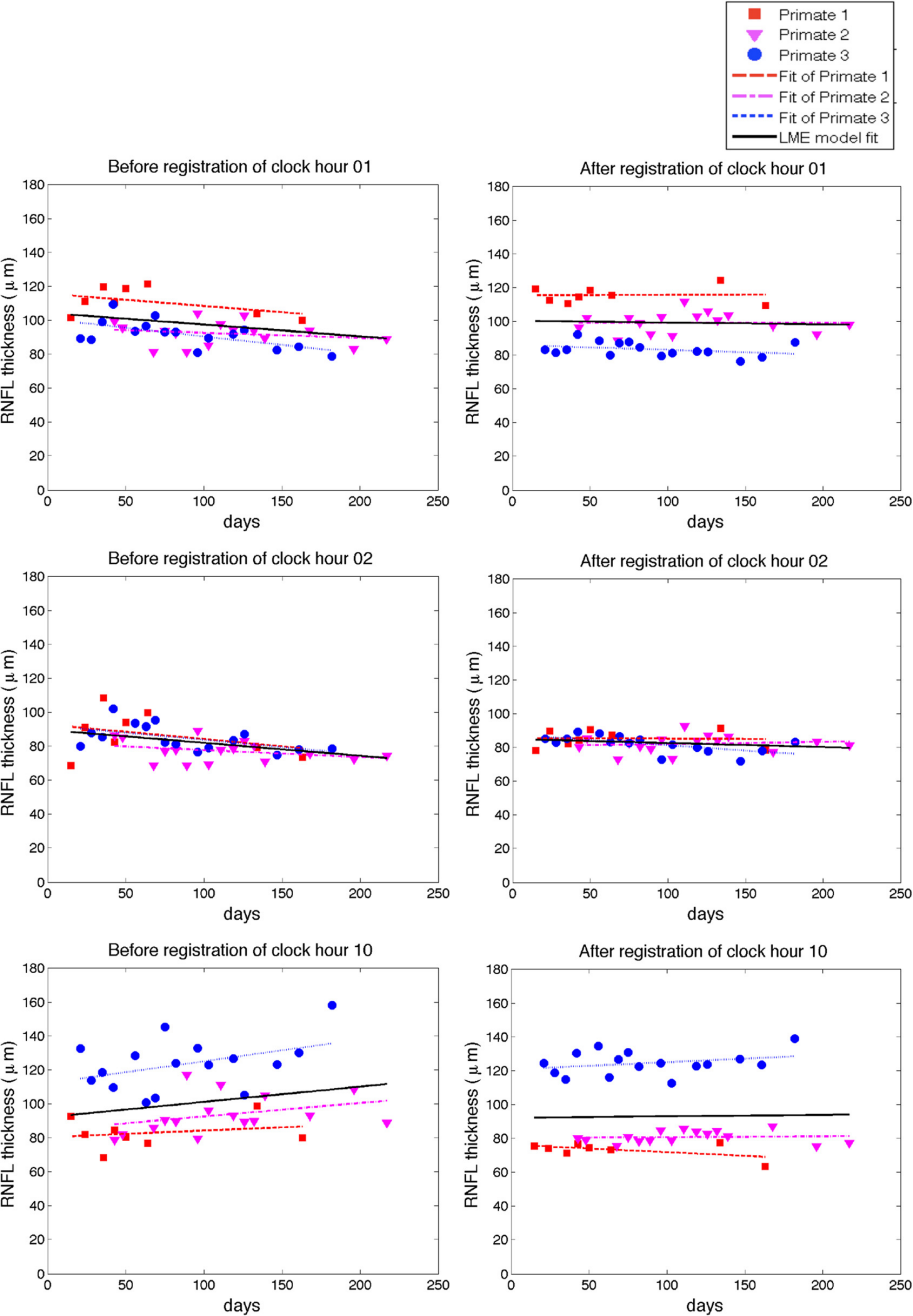
**Estimation of changes of RNFLT of clock hour 1, 2 and 10 average over time.** The left column is before registration, right column is after registration. The dashed lines are the fits of individual primates. The individual fits display very similar trends as compared to the linear mixed effects model fits. The clock hour 1 and 2 averages decreased significantly before registration (p = 0.0058 for clock hour 1 and p = 0.0054 for clock hour 2), but stayed constant after registration. The clock hour 10 average RNFLT increased significantly before registration (p = 0.0298), but was constant after registration.

We used a linear mixed effects model (Equation 7) to evaluate whether changes in RNFLT features occurred during the study duration. Before registration, RNFLT features are calculated in each map at each date. After registering all target images to a corresponding reference image using the LPCC_MSBV algorithm, we co-aligned all RNFLT maps, and used the overlapped region of all RNFLT maps from different dates to calculate the RNFLT feature parameter values. We found that prior to registration, three RNFLT features (1, 2, and 10 clock hour sectors averages) out of seventeen features we evaluated showed significant change during the study (Figure 7 and Table 4). Before registration, one and two o’clock hour sectors average RNFLT showed a significant decrease (p = 0.0058 for one o’clock hour and p = 0.0054 for two o’clock hour). Before registration, ten o’clock hour sector average increased significantly during the study duration (p = 0.0298). Other RNFLT features showed no change over the study duration. However, after registration, all RNFLT features, all model slopes of RNFLT feature vs. time are not significantly different from zero, suggesting that all thickness feature parameter values are constant over the time course of the study (Table 4). Since for healthy eyes, we would not expect the RNFL thickness to change significantly during the six months study duration [28], we concluded that consistency of RNFLT feature parameters improved after registration. The results suggest that registration can remove artifacts introduced by misalignment of RNFLT maps especially in more detailed features like 12 o’clock hour sector average. Overall, the clock hour features of RNFLT are more sensitive to mis-registration artifacts compared to the all-rings average and TSNI quadrants average.

**Table 4:** **Comparison of changes in RNFL features over time before and after registration**

**RNFL feature**	**Before registration**			**After registration**		
	**Intercept (μm)**	**Slope (μm/day)**	**p value for slope**	**Intercept (μm)**	**Slope (μm/day)**	**p value for slope**
**All rings**	102.00393	−0.00257	0.8232	101.0946	0.0024	0.8543
**Temporal**	81.6539	0.0378	0.1412	83.1664	−0.0085	0.5937
**Inferior**	132.2164	−0.0081	0.6857	132.5075	0.0030	0.8902
**Nasal**	82.7397	−0.0167	0.4794	74.1178	0.0105	0.4744
**Superior**	112.1748	−0.0280	0.2417	110.8570	0.0030	0.8702
**Clock hour 01**	104.2529	−0.0684	0.0058*	100.3047	−0.0105	0.5423
**Clock hour 02**	89.6372	−0.0764	0.0054*	85.0136	−0.0238	0.1335
**Clock hour 03**	75.1912	−0.0156	0.4824	67.9575	0.0081	0.5795
**Clock hour 04**	83.2301	0.0345	0.4629	69.5369	0.0295	0.0806
**Clock hour 05**	129.4731	0.0205	0.5714	119.3772	0.0059	0.7964
**Clock hour 06**	141.3183	−0.0032	0.9131	144.1395	0.0044	0.8891
**Clock hour 07**	126.2726	−0.0456	0.2689	134.3108	−0.0003	0.9919
**Clock hour 08**	80.2741	−0.0248	0.4117	87.8058	−0.0183	0.3725
**Clock hour 09**	72.7352	0.0491	0.2124	69.7586	−0.0149	0.3827
**Clock hour 10**	92.0908	0.0901	0.0298*	92.1486	0.0085	0.6408
**Clock hour 11**	115.7404	0.0270	0.4125	113.4984	0.0235	0.3103
**Clock hour 12**	116.1991	−0.0349	0.2496	116.8135	−0.0010	0.9574

A linear mixed effects model is used to estimate the change in RNFL features over time. Coefficients and the p values of the coefficients of the linear mixed effects model of RNFL features vs. days before registration and after registration by LPCC_MSBV algorithm are shown in this table. The intercept represents the magnitude of RNFL thickness and, therefore, the p values for intercepts are all zero indicating that the thickness is non-zero. The slopes represent the change of RNFL thickness features over time. The p values for the slopes are shown in the table.

*p values were smaller than 0.05.

Moreover, we also compared the residuals of the linear mixed effect model before registration and after registration using the LPCC_MSBV algorithm (Table 5). We found that for most RNFLT features, the magnitude of residuals of the linear mixed effects model were significantly decreased after registration (slopes were negative and p values were smaller than 0.05; marked with “*” in Table 5). Therefore, registration reduces the measurement error.

**Table 5:** **Comparison of the magnitude of residuals of the linear mixed effect model before and after registration**

**RNFL feature**	**Linear mixed effects model for residual**		
	**Intercept (μm)**	**Slope Of** ***reg***_***i,t***_	**p value for slope**
**All rings**	2.5198	0.3877	0.4075
**Temporal**	5.3737	−1.8789	0.0425*
**Inferior**	4.5483	0.0420	0.9595
**Nasal**	5.1851	−1.8431	0.0259*
**Superior**	5.6397	−1.4297	0.0773
**Clock hour 01**	5.3477	−1.3711	0.0944
**Clock hour 02**	6.0659	−1.9699	0.0359*
**Clock hour 03**	4.7984	−1.3750	0.0797
**Clock hour 04**	9.3879	−6.3046	<0.0001*
**Clock hour 05**	8.7581	−3.5554	0.0018*
**Clock hour 06**	6.5226	0.6532	0.5933
**Clock hour 07**	8.0791	−2.8944	0.0325*
**Clock hour 08**	6.5612	−2.2646	0.0488*
**Clock hour 09**	7.6976	−4.3798	0.0024*
**Clock hour 10**	8.7744	−4.9544	0.0003*
**Clock hour 11**	7.6400	−2.6737	0.0229*
**Clock hour 12**	7.1031	−2.6395	0.0060*

Coefficients and the p values of the coefficients for comparing residuals of linear mixed effects models of RNFL features vs. days before registration (*reg*_*i,t*_ = 0) and after registration by LPCC_MSBV algorithm (*reg*_*i,t*_ = 1). Most of the slopes are significantly negative (p < 0.05), which mean that the magnitude of the residuals decreased after registration.

*Slopes were negative and p values were smaller than 0.05.

## Conclusions

In this study, we investigated benefits of image registration on estimation of longitudinal RNFLT changes in non-human primate eyes. We compared the performance of MI and LPCC_MSBV algorithms. Precision and recall calculated between manually segmented blood vessel image pairs were used for comparison with that determined after applying LPCC_MSBV and MI algorithms. Results indicate that application of either MI or LPCC_MSBV algorithms improves the alignment between target and reference images compared to no registration. The precision after registration by the LPCC_MSBV algorithm is significantly higher than that after registration by the MI algorithm. Recalls following registration by either MI or LPCC_MSBV algorithms are similar. The computation time of the LPCC_MSBV algorithm was five-times faster than that of the MI algorithm. However, this computation time does not include the pre-processing time required to manually segment the blood vessels before application of the LPCC_MSBV algorithm. Therefore, when fully automated registration is required, MI is preferred to LPCC_MSBV algorithm. Both MI and LPCC_MSBV algorithms showed good performances for registration of fundus images of primate eyes and thus have potential for application to OCT image data recorded from human eyes.

The present study is the first to evaluate how registration can affect the analysis of RNFLT measurement in a longitudinal study on healthy eyes using a non-human primate model. We evaluated the registration effect on all reported RNFLT feature parameters, which includes all-rings average, TSNI quadrants average, and 12 o’clock hours average. The results suggest that RNFLT feature parameters evaluated in the 12 o’clock hours are affected by registration in a longitudinal study in healthy primate eyes. Some recent studies also supported the observation that RNFLT average in some clock hour sectors are more sensitive to head tilt or OCT instrument variability [29,30]. Registration can correct the artifacts introduced by misalignment of RNFLT maps recorded on different dates. Registration allows detection of changes of detailed features and prevents false detection of changes due to misalignment. Moreover, any analyses associated with the all-rings average and TSNI quadrants average are not affected by the registration. Misalignment of a series of RNFLT maps is a candidate reason that previous studies showed that the all-rings average is the most robust feature in reproducibility studies [4,31]. Our results suggest the 1, 2, and 10 clock hour sectors are the most sensitive to registration errors possibly because these clock hour sectors are located in regions with a large RNFLT gradient. Intuitively, sectors that are in RNFLT gradient transition zones should be more sensitive to mis-registration than sectors in smooth areas of RNFLT maps. Therefore, without registration, the variations of RNFLT features across different dates are due to misalignments among RNFLT maps plus the reproducibility error introduced by the instrument. With registration, the variations of RNFLT features across different dates are primarily due to the reproducibility error introduced by the instrument.

This study was performed on non-human primates. Due to the difference in eye fixation method during imaging acquisition, primate experiments magnify rotation artifacts because of the suture positioning process that was performed to bring the primate’s ONH into the center of the field of view. In a clinical setting where a patient can fixate on a target, human eyes may have smaller rotation variation from one imaging session to another. However, human eyes can still exhibit comparable translation factors vs. primate eyes because this effect is primarily due to the variability of operator’s placement of the scanning ring around the ONH. Implementing registration algorithms for OCT images has the potential to improve analysis and interpretation of evolution of spatial changes of RNFLT over time as assessed in this longitudinal study. Results of such longitudinal studies can potentially identify features of RNFLT that precede visual field changes and allow for earlier and more effective therapeutic interventions.

## Additional file

Additional file 1: Table S1. Comparisons of PS-OCT, RTVue OCT and Cirrus OCT systems.

## Abbreviations

RNFLT: Retinal nerve fiber layer thickness
ONH: Optic nerve head
OCT: Optical coherence tomography
MI: Mutual information
LPCC_MSBV: Log-polar transform cross-correlation using manually segmented blood vessels
TSNI: Temporal, superior, nasal and inferior
