# Supplementary material for: Effect of image registration on longitudinal analysis of retinal nerve fiber layer thickness of non-human primates using Optical Coherence Tomography (OCT)
Source: Eye Vis (Lond). 2015 Feb 12;2:3. doi: 10.1186/s40662-015-0013-7 (PMC4657366; doi:10.1186/s40662-015-0013-7)
Supplement: Additional file 1: Table S1. — Comparisons of PS-OCT, RTVue OCT and Cirrus OCT systems. [file 40662_2015_13_MOESM1_ESM.docx]

**Additional file 1**

**Table S1. Comparisons of PS-OCT, RTVue OCT and Cirrus OCT systems.**

| Instruments | Input Light Wavelength | A-Scan Rate | Axial resolution | Transverse resolution | Scanning Pattern |
| --- | --- | --- | --- | --- | --- |
| PS-OCT | 1064nm | 34000 A-scans/s | 12μm | 25 μm | ring |
| RTVue OCT | 840nm | 26000 A-scans/s | 5 μm | 15 μm | ring/ radial |
| Cirrus OCT | 840nm | 27000 A-scans/s | 5 μm | 15 μm | raster |
